# The State Medical Association’s Efforts at Medical Legislation

**Published:** 1906-06

**Authors:** 


### TEXAS MEDICAL JOURNAL.

AUSTIN, TEXAS.

A MONTHLY JOURNAL OF MEDICINE AND SURGERY.

EDITED AND PUBLISHED BY
F. E. DANIEL, M. D.
Mrs. F. E. DANIEL, Business Manager.

Published Monthly at Austin, Texas. Subscription price $1.00 a year in advance

The practice of medicine will be very much as you make it—to one a worry, a care, a perpetual annoyance; to another, a daily joy and a life of as much happiness and usefulness as can well fall to the lot of man.—*Osler.*

### THE STATE MEDICAL ASSOCIATION'S EFFORTS AT MEDICAL LEGISLATION.

### Senator Davidson's Relation Thereto.

Senator A. V. Davidson, of DeWitt county, candidate for Lieutenant Governor, has issued an address to the people of Texas stating his platform; or, as it is headed, "Where He Stands." In that address (I clip from the *Daily Tribune*, Austin), he says:

"Believing in the scientific treatment of disease, I introduced and we passed the medical association's bills; my purpose in so doing being to protect the public against unqualified practitioners of medicine, and also protect the profession against the same class of people. There has been no matter before the people of Texas more vital to their interest, since I have been a member of the senate, than the public health."

I can not, in justice to the medical profession of Texas, let this claim go unchallenged. The claim is not borne out by the Senate record; nor does it tally with the recollection of the writer and his associates on the State Medical Association's committees representing the Association before the past several Legislatures and who were in constant attendance.

Senator Davidson is a courteous and accomplished gentleman, albeit a little erratic and irascible,—an earnest and forceful speaker, and he did champion the cause of legitimate medicine at least at one session, but *failed.* The drugless element, including the Christian Scientists, were too much for us. We have to thank him for that effort, but not for any bills he introduced, and "we" passed.

In order that the physicians of Texas, who, for twenty years have striven to procure medical legislation in the interest of the public health, may understand the nature and extent of the Senator's services and appreciate their real obligations (?) to him, I give, as briefly as is consistent with clearness, a resume of the efforts at medical legislation before the Legislatures of 1901, 1903, and 1905.

In 1901 the State Medical Association prepared a bill to regulate the practice of medicine by means of a State Board of Medical Examiners. Dr. J. T. Wilson, of Sherman, chairman of the Legislative committee, placed it in the hands of Judge Walker, of Grayson county, member of the House, who, for a week or more, made a gallant fight for its passage. The drugless element—including osteopaths and Christian Scientists; and the homeopaths, *et id. om. gen.*, were there in full force, and the bill came out so mutilated that its parents didn't know it. Recognition was given the homeopaths and the eclectics, and, instead of one board of examiners, asked for, three boards were created, one for the regular profession, and one each for the homeopaths and eclectics; and a clause was inserted, exempting, altogether, from the provisions of the act, the Christian Scientists and all others of the drugless persuasion, *id est*, "all who do not give drugs or medicines,"—the faith-healers, the magnetic fellows, the osteopaths, and Dr. Sam,—the Voodoo doctor. Thus, it will be seen they whipped us out in the House. Score one for the drugless and the Ch. Sci.

The question to be decided by the Association committee was: "Shall we put this amended bill through the Senate and let it go at that? or, shall we introduce the original bill in the Senate and fight it all over again?" A consultation was held by the committee and it was agreed to accept the bill as it came from the House,—the three boards and the outrageous exemption clause, and trust to the future to get that clause stricken out. The bill was the special order in the Senate one day for 3 o'clock. There was a woman Christian Science disciple seated by about every third Senator, scores of male representatives of that sect were present, and osteopaths galore, ready to jump on the original bill with both feet. Dibrell was loaded, and had on his war-paint, ready to do and dare and die, if need be, in defense of Christian Science. They bluffed us. Word was sent to Dibrell to lay down his tomahawk; that *we*

*conceded his contention as to exempting the Ch. Sci.* It was oil on the troubled waters. The House bill (exempting osteos, magnetic healers, and Ch. Sci.) was introduced (by Davidson, I think), referred to committee, reported favorably, read three times, and passed, all within fifteen minutes. Not a gun was fired nor a word spoken. That is the law now on the statute books. Surely Mr. Davidson does not claim any credit for it. If there be any "credit" —in forcing upon us a most unjust and obnoxious bill, Judge Walker is entitled to it. We surrendered to the enemy. Score 2 for the Ch. Sci. and the osteos.

At the session of 1903 the State Association sought, through its committee (Daniel, chairman) to knock out the exemption clause, and Senators Davidson and Hicks made a strenuous, *but ineffectual, fight.* The enemy were in full force, and there was so near a tie on count, that rather than suffer *defeat,* Mr. Davidson sent the bill back to committee (and killed it, of course). It was a dog fall. Score 3 for the Ch. Sci. and osteos. Any credit in *that?*

[I should state here, parenthetically, two things: First, prior to our reorganization the committee in charge of legislation was called "The Committee on State Board of Health." In 1903-4 it was changed to "Committee on Public Policy and Legislation." Second, at the 1903 session of the Legislature a Bill for a State Board of Health (in addition to the bill to knock out the exemption clause of the Practice Act) was drawn up by the committee, which consisted of Daniel, chairman; Paschal, Blalock, Saunders, Coleman, and Tabor (Tabor had been added by request of the chairman). Two of the members, Paschal and Blalock, objected to it, and it was abandoned; and in lieu thereof a bill "To Enlarge the Powers of the Quarantine Department and Change Its Name to Department of Public Health and Vital Statistics" was drawn up and agreed to by Daniel, Tabor, Coleman, and Saunders, and it was introduced by Senator *Harper* at Dr. Tabor's request. It went to committee, was reported favorably, was special order for 3 o'clock in the Senate. There was no objection, and everything seemed favorable to its speedy passage. At 3 o'clock two members from the West Texas Medical Society, Drs. Paschal and Burleson, appeared in the Senate, and, without the knowledge or consent of Drs. Tabor and Daniel, had Senator Hicks, of Bexar, to call off the bill. It was re-referred to committee—and *killed.* Two fragments of it were rescued, one changing the name of the Quarantine Department, and one providing for registration of vital statistics. These passed, the vital statistics bill, *with the appropriation*

*stricken out*—emasculated. Any credit here? If so, Harper is entitled to it.]

And now comes the third attempt at regulating the practice, and the second attempt to strike out the unjust exemption clause (session 1905). Hicks deserted us and went over to the osteos, and notified the committee of his change of heart. Daniel was again chairman of committee (ex-officio, as he was President of the Association). No other member of the committee was at Austin, and he called to his assistance Drs. M. M. Smith and J. S. Wooten, of Austin, as auxiliary members of the committee. The bill striking out the exemption clause was introduced in March by Senator Davidson, was referred to his own committee (Judiciary No. 1), and representatives of all the so-called "schools of medicine" were present before the committee. The Christian Science people offered an amendment exempting that sect from examination by a medical board, as they do not pretend to medical knowledge, and every examination meant rejection, of course,—the amendment obligating every Christian Scientist, under penalty to be fixed by the bill, to not undertake the treatment of contagious diseases, surgical or obstetrical cases; but, under penalty, also, for failure to do so,—to call a licensed practitioner to all such cases coming to their hands.

Dr. Daniel agreed to the amendment, as just, right and proper, inasmuch as the proposed proscription—the examination of these people in the branches of medicine meant *prohibition,* and said that the people of Texas would not consent to the utter crushing out by law, of a cult—believed in by thousands of educated and good people. Moreover, he showed that letting the Christian Science people alone, after tying their hands as to contagious diseases, surgery, and obstetrics, was demanded as expedient, seeing that they had, with their collaborators—the drugless element—defeated us in the House in 1901, bluffed us in the Senate in 1901 into an agreement with them and Dibrell to let them alone, utterly defeated us in the Senate in 1903, when the fight was led by Davidson and Hicks, resulting in a dog fall, Davidson not letting it come to a vote. By disarming the Christian Scientists of the power for harm, and letting them alone, it was argued that their opposition to our bill would cease; indeed, we were assured of their assistance in getting the clause knocked out as to the other fellows. Dr. Wooten agreed to the proposed amendment and followed Dr. Daniel in the same line of argument. Dr. Smith remained silent and refused to either consent or object. Senator Davidson, chairman of the Senate committee and in charge of the Association's bill, and to whom we looked to champion our cause, alone, as Hicks had forsaken us, became very angry, and throwing the bill down, said, "I'll have nothing more to do with it." He then gave vent to a bitter denunciation of all and singular the whole Christian Science element. Any credit coming to him here? Had that amendment been adopted and the bill reported favorably, it is believed that Mr. Davidson could have put it through.

Then came the State Medical Association's bill to devote the unclaimed cadavers from the State's charity hospitals to scientific uses—in furtherance of the essential basis of medical education and science,—a knowledge of anatomy. This bill was written by the faculty of our great medical school; its passage was asked for by the president and faculty of the great University of Texas, was referred to the State Medical Association; was approved by its House of Delegates, and ratified by the entire body; was placed in the hands of the President of the Association, ex-officio chairman of the Committee on Medical Legislation, and by him and his associates placed in the hands of Senator Looney, who introduced it in the Senate.

I would gladly throw the mantle of charity over the disgraceful scenes that followed, especially as it has become so notorious, and I would do so but for the absurd claim that the distinguished gentleman gives to the daily press in furtherance of his candidacy for Lieutenant Governor,—a claim which I have shown has no real basis. I dare say that he and Senator Chambers—who exclaimed, "If Jesus Christ were crucified today, the doctors of Texas would steal the body before night,"—and Senator Hanger, who stigmatized the doctors of Fort Worth as "most ungodly butchers," can hardly contemplate their share in the slaughter of this bill with pride and satisfaction or with that calm equanimity that should characterize a statesman. And, lest we forget, I here remind my esteemed fellows of the Association that it was their champion,—he who "introduced and we passed the State Association's *bills*" who moved to strike out "pauper" and insert "doctor," adding that it was "no jest"; he "meant it." "Let them dissect each other," he said. Any credit coming to him here?

And yet, he "believes in the scientific treatment of diseases" and appreciates "the importance of protecting the public health."

The bill was kicked out of the Senate. It was not accorded the courtesy of an intelligent discussion nor a vote. It was laughed at, scorned, and treated with the utmost indignity.

Such a law would have done more to protect the sanctity of the family burying-ground than all else combined; for, medical colleges must have anatomical material, and so long as there is a cash demand for cadavers, there are ghouls who will furnish them; hence, grave-robbing.

The State requires doctors to know anatomy.

